# Myeloid sarcoma presenting as an isolated pancreatic mass in a 3‐year‐old child

**DOI:** 10.1002/jpr3.70192

**Published:** 2026-05-07

**Authors:** Jappmann Kaur Monga, Cara L. Mack, Michelle Saad, Dylan Applin, Maisam Abu‐El‐Haija, David Vitale, Zachary Graff, Gila Ginzburg

**Affiliations:** ^1^ Department of Pediatrics, Division of Pediatric Gastroenterology, Hepatology & Nutrition Medical College of Wisconsin Milwaukee Wisconsin USA; ^2^ Department of Pediatrics, Division of Gastroenterology, Hepatology & Nutrition, Cincinnati Children's Hospital Medical Center Cincinnati College of Medicine Cincinnati Ohio USA; ^3^ Department of Radiology, Children's Wisconsin Medical College of Wisconsin Milwaukee Wisconsin USA; ^4^ Department of Pediatrics, Division of Pediatric Hematology, Oncology and Stem‐Cell Transplantation Medical College of Wisconsin Wauwatosa Wisconsin USA

**Keywords:** endoscopic retrograde cholangiopancreatography (ERCP), endoscopic ultrasound, extramedullary tumor, obstructive jaundice

## Abstract

Myeloid sarcoma (MS) is an extramedullary tumor of myeloid precursor cells, frequently associated with acute myeloid leukemia (AML), and rarely occurring in isolation. We present a child with obstructive jaundice secondary to a pancreatic head mass. Initial imaging was consistent with pancreatic hematoma; however, continued symptoms led to endoscopic ultrasound (EUS)‐guided biopsies that raised concern for autoimmune pancreatitis. Further histological and cytogenetic analysis confirmed pancreatic MS associated with a RUNX1::RUNX1T1 fusion. Bone marrow evaluation was negative by conventional diagnostic methods; however, reverse transcriptase with qualitative real‐time polymerase chain reaction (RT‐qPCR) detected RUNX1::RUNX1T1 transcripts. Chemotherapy achieved both radiologic resolution and disappearance of RUNX1::RUNX1T1 transcripts within the bone marrow. This represents one of the first reported cases of isolated pancreatic MS in a child, without progression to AML. Our case highlights the diagnostic challenges of pancreatic masses, the underutilization of EUS‐guided biopsy in pediatrics, and the use of RT‐qPCR for both diagnosis and disease resolution.

## 1 INTRODUCTION

Myeloid sarcoma (MS) is an extramedullary solid tumor of myeloid precursor cells, typically associated with acute myeloid leukemia (AML) or other myeloid neoplasms, but rarely occurs in isolation.^1^ Isolated MS, defined by the absence of leukemic blasts on blood smears and bone marrow, has a reported adult incidence of 2 per 1,000,000, with unknown incidence in pediatrics due to rarity, but likely even lower.^2^ Untreated MS will progress over 5–12 months to AML in >90% of cases.^3^ MS can occur in the skin, lymph nodes, bone, and less commonly other organs, including the pancreas.^1^ MS in the pancreas and hepatobiliary tract is extremely rare, especially in children. Pancreatic MS presents similarly to other pancreatic masses: obstructive jaundice secondary to compression of the extrahepatic biliary system, exocrine pancreatic insufficiency secondary to compression of the pancreatic duct (PD), and other vague symptoms including abdominal pain. Diagnosis requires tissue biopsy, often through open laparoscopy; however, there is growing use of EUS and endoscopic retrograde cholangiopancreatography (ERCP).^1^ There is often a delay in MS diagnosis due to its rarity, which is further compounded in children due to limited availability of EUS/ERCP. Here we present a 3‐year‐old male with obstructive jaundice who was found to have pancreatic head MS.

## 2 CASE REPORT

A 3‐year‐old previously healthy male presented with scleral icterus, jaundice, pruritus, acholic stools, abdominal pain, and lethargy. Parents denied recent travel, fevers, or rashes, but noted sustained mild abdominal trauma 4 weeks prior. Initial labs: aspartate aminotransferase 1230 U/L (8–60 U/L], alanine transaminase 1205 U/L (<29 U/L), total bilirubin 5.3 mg/dL (<1.0 mg/dL), direct bilirubin 2.2 mg/dL (<0.3 mg/dL), gamma‐glutamyl transferase 1914 U/L (<21 U/L), alkaline phosphatase 2918 U/L (335–450 U/L), alpha‐fetoprotein 1.7 ng/mL (<40 ng/mL), and lipase 967 U/L (10–120 U/L). Liver ultrasound reported hepatosplenomegaly and dilatation of the common bile duct (CBD) (6–7 mm). Magnetic resonance cholangiopancreatography (MRCP) confirmed CBD/PD dilatation and revealed a 4 × 3.3 × 2.6 cm intrinsically T1 hyperintense, non‐enhancing pancreatic head mass concerning for hemorrhage (Figure 1A,B). Symptoms and liver tests improved spontaneously, thought due to resolving pancreatic hematoma.

**Figure 1:**
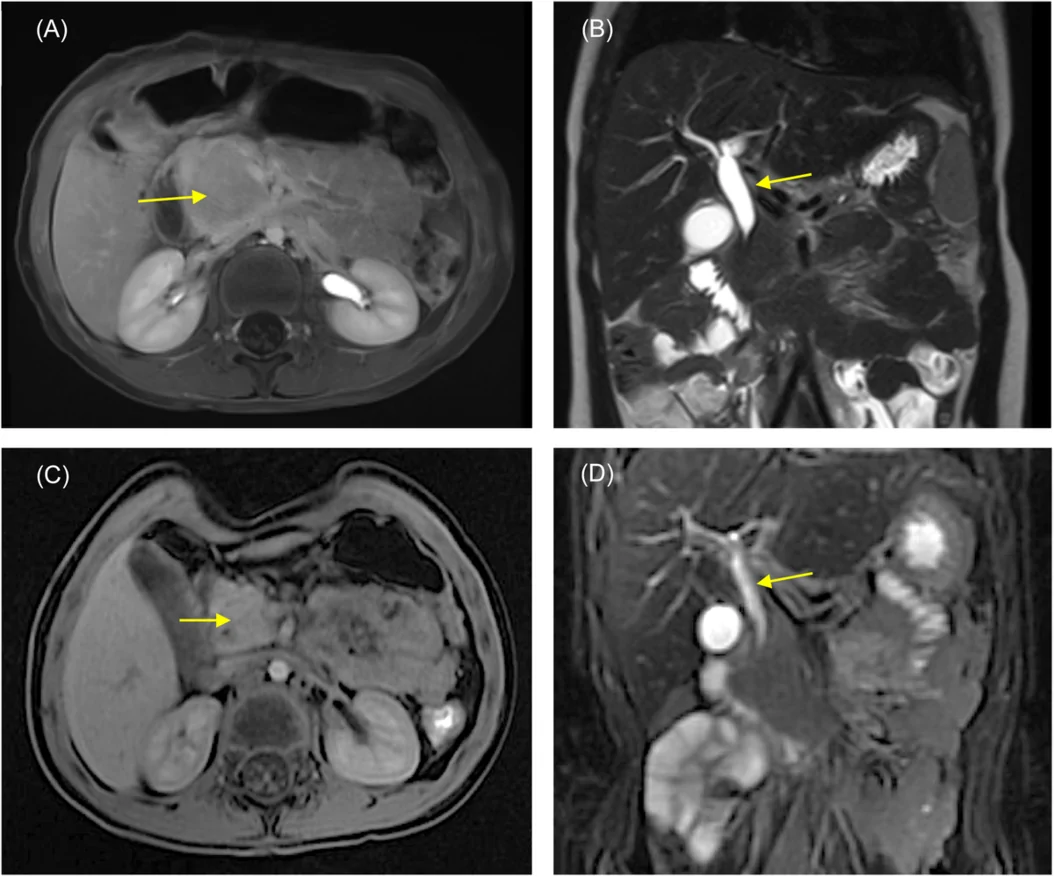
Select MRCP images of the abdomen, pre‐ and post‐chemotherapy. (A) Axial T1 fat‐saturated, contrast‐enhanced image showing lobulated, hypo‐enhancing mass in the pancreatic head measuring 4 cm (arrow) and displacing adjacent structures; (B) Coronal T2‐weighted image showing dilated common bile duct (arrow); (C) Post‐chemotherapy axial T1 fat‐saturated image demonstrating normal‐sized pancreatic head with T1 signal isointense to that of the remaining pancreas and absence of a defined mass; (D). Post‐chemotherapy coronal T2 fat‐saturated image demonstrating decreased common bile duct dilatation. MRCP, magnetic resonance cholangiopancreatography.

Two weeks later, transaminases, bilirubin and lipase (3373 U/L) rose; fatigue and poor intake worsened. Repeat MRCP demonstrated a similar‐sized, lobulated, mass‐like lesion with slight worsening of CBD/PD dilatations. An adult advanced endoscopist performed an EUS, revealing bile duct dilatation (8–9 mm), PD dilatation (3 mm) and severe distal bile duct stricture. EUS‐guided fine needle biopsies and ductal brushings were non‐diagnostic. ERCP with sphincterotomy and biliary stent placement was performed, but computed tomography (CT) later showed stent tenting, prompting removal.

Second opinion and repeat MRCP performed 15 days subsequently showed patchy T1 hyperintensity and T2 isointensity raising suspicion for focal autoimmune pancreatitis (AIP). ERCP‐guided CBD stent placement and repeat EUS‐guided biopsies of the pancreatic mass initially reported AIP (Figure 2); steroids were initiated. Immunohistochemistry later revealed dysplastic myeloid cells and *t*(8;21)(q22;q22) RUNX1::RUNX1T1 rearrangement, confirming MS. Steroids were promptly discontinued after 5 days; pediatric oncology evaluation followed. Bone marrow biopsy was morphologically negative for AML, however, 1% of cells were positive for the RUNX1::RUNX1T1 fusion via fluorescence in situ hybridization (FISH) and RT‐qPCR. positron emission tomography (PET) showed uptake only in the lesion. Standard induction chemotherapy (cytarabine, daunorubicin, gemtuxumab) was initiated.^4^ After induction 1, marrow was negative by FISH and RT‐qPCR; but MRCP showed persistent, smaller mass. Induction 2 (fludarabine, cytarbine, idarubicin) achieved complete resolution and decreased CBD dilatation (Figure 1C,D). He completed three consolidation cycles (total 5) and remains in remission 6 months off therapy.

**Figure 2:**
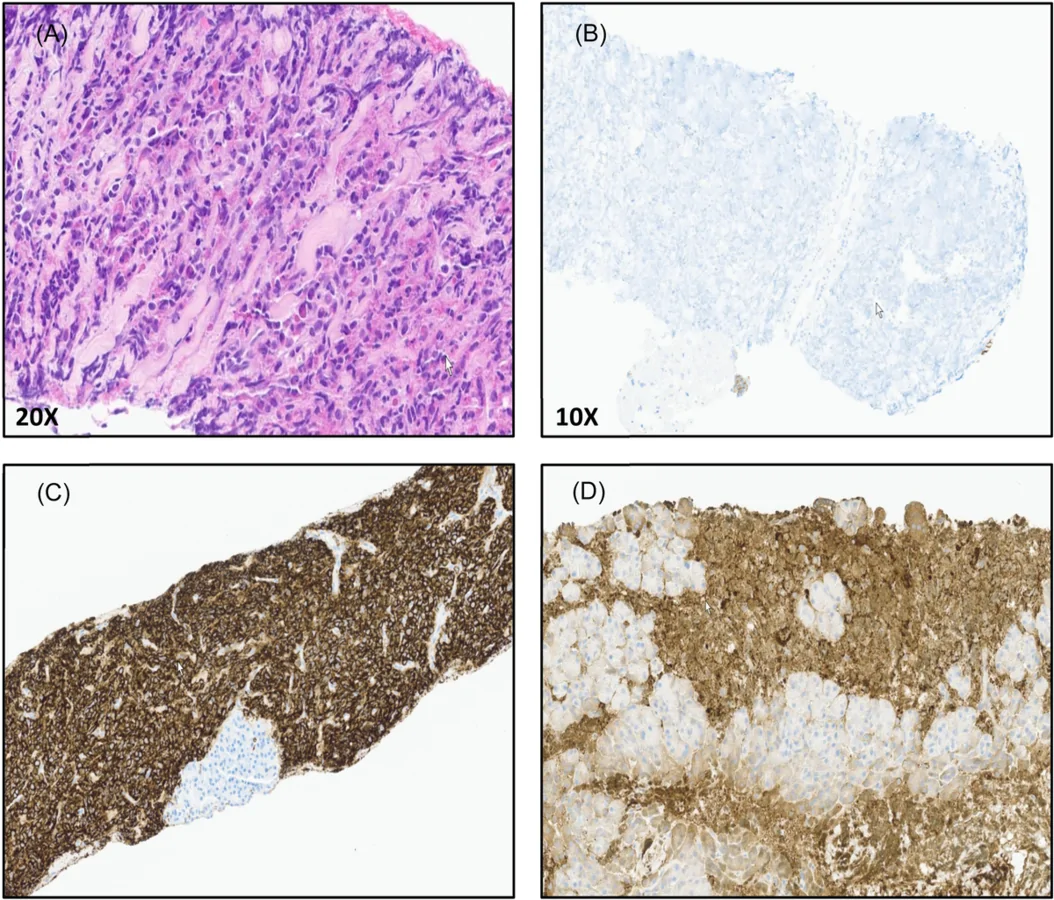
Histology of the pancreatic mass. (A) H&E staining showing eosinophilic granules; (B) Negative CD138 immunohistochemical staining, indicating absence of plasma cell infiltrations commonly associated with autoimmune pancreatitis; (C) Positive CD43 staining (brown) raises possibility of atypical lymphoid or myeloid cell involvement; (D) Positive MPO staining (brown), supporting presence of myeloid lineage cells. H&E, hematoxylin and eosin; MPO, myeloperoxidase.

## 3 DISCUSSION

MS in children remains poorly understood, presenting unique diagnostic and management challenges.^5^, ^6^ Literature reports only four pediatric cases with obstructive jaundice from pancreatic MS with AML^7^, ^8^, ^9^ (Table 1). Compared to these, our case is among the first descriptions of isolated pancreatic MS in a child. Given the, there is minimal pediatric‐specific literature to guide diagnosis and decision‐making. Adult guidelines target patients with different comorbidities and life expectancies. Imaging findings may also differ from adults. Our patient's initial MRCP different from prior pediatric cases; MS typically appears T2 hyperdense, T1 isointense with homogenous enhancement,^5^ but ours was intrinsically T1 hyperintense.

**Table 1:** Summary of prior case reports of myeloid sarcoma, detailing mass location, imaging findings, and diagnostic methods.

	Author	Age (years)/gender	Clinical presentation	MS location	Diagnosis
1	Jappmann et al. [current case report]	3/M	Obstructive jaundice	Head of pancreas	Isolated MS
2	Jaing et al.^7^	4/M	Jaundice, RUQ pain, pallor, hepatomegaly	Head of pancreas	AML
3	Mwanda et al.^8^	3.5/M	Jaundice, pallor, anorexia, emesis, fever, abdominal distention	Pancreas	AML
4	Miri‐Aliabad et al.^9^	3/F	Obstructive jaundice	Pancreas	AML
5	Alsolami et al.^10^	12/M	Obstructive jaundice, episodic epigastric pain	Head of pancreas	Isolated MS

Abbreviations: AML, acute myeloid leukemia; F, female; M, male; MS, myeloid sarcoma; RUQ, right upper quadrant.

Our case highlights the utility of EUS and ERCP for diagnosis and ductal obstruction management. These modalities are particularly valuable in pediatric patients, offering high‐resolution imaging along with diagnostic and therapeutic capabilities through a minimally invasive approach. No consensus exists on endoscopic appearance of MS versus other pancreatic malignancies. In our case, the lesion was lobulated with well‐demarcated borders, unlike pancreatic adenocarcinoma. Diagnosis was challenging as imaging suggesting hematoma or AIP, and initial histology report AIP. Definitive diagnosis occurred only after identifying the RUNX1::RUNX1T1. While steroids can alter morphology of biopsy specimens, this is much less pronounced in AML/MS compared to ALL. Given this and that the course was short, it did not delay diagnosis or alter prognosis. While marrow was negative morphologically, measurable residual disease testing detected the fusion, enabling sensitive treatment response tracking and guiding imaging follow up for mass resolution.

## 4 CONCLUSION

Pancreatic MS should be considered in the differential diagnosis of isolated pancreatic masses in children. Further, our case supports the utility of pediatric EUS‐guided diagnostic biopsies, allowing for the successful initiation of chemotherapy to cure this aggressive disease, prior to the onset of AML.

## CONFLICT OF INTEREST STATEMENT

The authors declare no conflicts of interest.

## ETHICS STATEMENT

Informed parental consent was obtained for publication of case details.
